# Supplementary material for: A remote-controlled automatic chest compression device capable of moving compression position during CPR: A pilot study in a mannequin and a swine model of cardiac arrest
Source: PLoS One. 2024 Jan 19;19(1):e0297057. doi: 10.1371/journal.pone.0297057 (PMC10798619; doi:10.1371/journal.pone.0297057)
Supplement: S1 Table — (DOCX) [file pone.0297057.s001.docx]

S1 Table 1. Specifications of ROSCER

| Product name | ROSCER |
| --- | --- |
| Compression rate | 90, 100, 110, 120/min |
| Compression depth | 4 - 6 cm |
| Size | 620 mm (Height) x 245 mm (depth) x 547 mm (width) |
| Degree of freedom | 3 |
| Weight (including battery) | 9.3 kg |
| Run time of battery | 60 min |
| Sternum height | 177 – 260 mm |
| Maximum chest width | 448 mm |
