# Supplementary figures and images for: A remote-controlled automatic chest compression device capable of moving compression position during CPR: A pilot study in a mannequin and a swine model of cardiac arrest
Source: PLoS One. 2024 Jan 19;19(1):e0297057. doi: 10.1371/journal.pone.0297057 (PMC10798619; doi:10.1371/journal.pone.0297057)

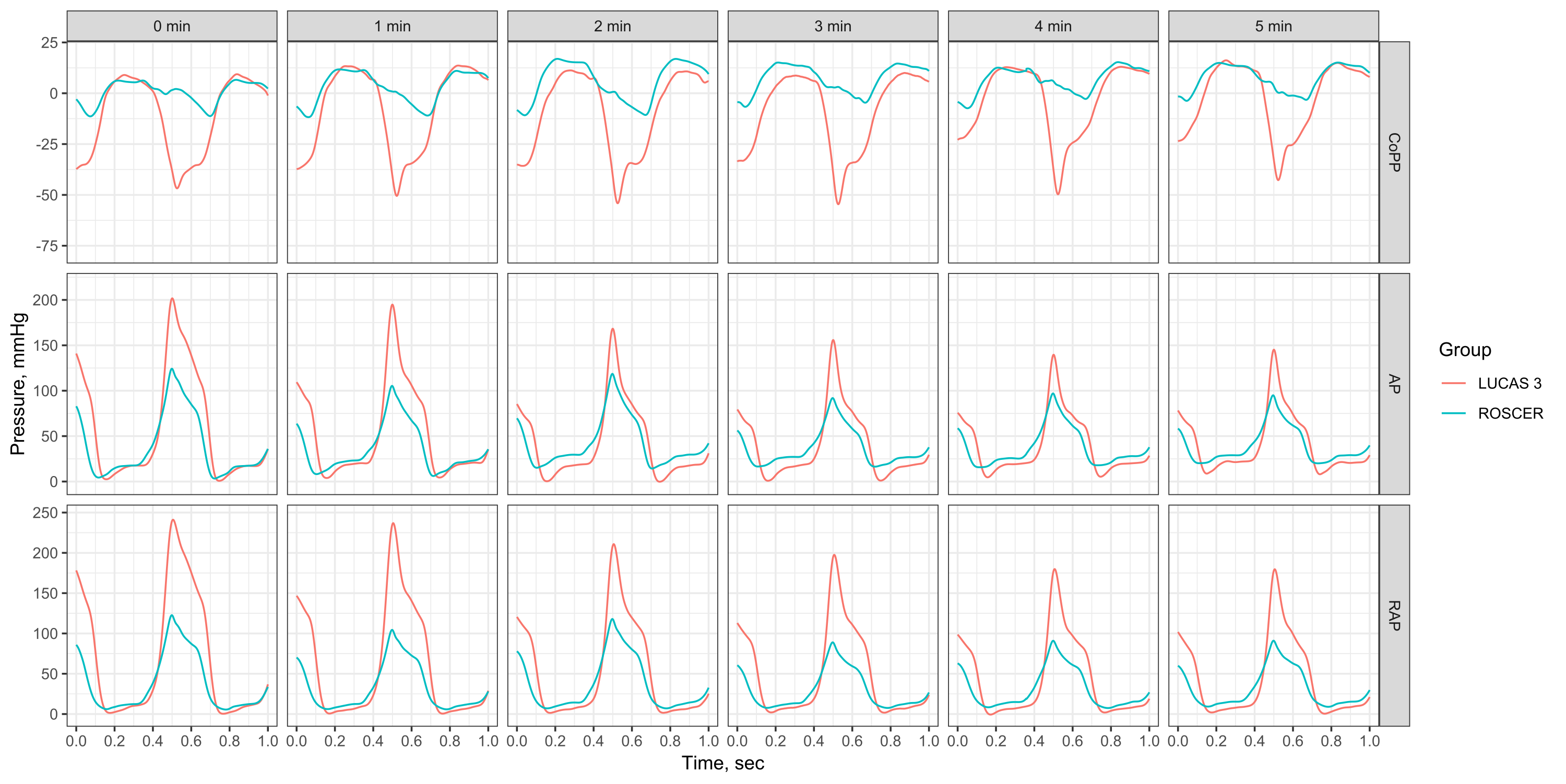

Supplement: S2 Fig — (TIFF) [file pone.0297057.s003.tiff]
